# Management of paroxysmal nocturnal hemoglobinuria with low-level hemolysis in pregnancy– a report of two cases

**DOI:** 10.1007/s00277-024-06086-z

**Published:** 2024-11-13

**Authors:** Julia Riedl, Michael Pfeilstöcker, Alex Farr, Günther Häusler, Cihan Ay, Wolfgang Füreder

**Affiliations:** 1 Department of Medicine I, Division of Hematology & Hemostaseology, Medical University of Vienna, Vienna, Austria; 2 3rd Medical Department for Hematology and Oncology, Hanusch Hospital, Vienna, Austria; 3 Department of Obstetrics and Gynecology, Division of Obstetrics and Feto-Maternal Medicine, Comprehensive Center for Pediatrics (CCP), Medical University of Vienna, Vienna, Austria

**Keywords:** Paroxysmal nocturnal hemoglobinuria, Thrombocytopenia, Pregnancy, Eculizumab, Anticoagulation

## Abstract

Pregnant women with paroxysmal nocturnal hemoglobinuria (PNH) are at high risk for life-threatening thromboembolism. Therapy with the complement inhibitor eculizumab is able to mitigate thrombotic risks in PNH and to improve pregnancy outcomes. However, whether PNH with low-level hemolysis in pregnancy can be safely managed without complement inhibition is unclear.

Here, we describe two pregnant patients with PNH in the setting of bone marrow failure and low-level hemolysis with lactate dehydrogenase (LDH) < 1.5 x upper limit of normal [ULN]. In both patients, management consisted solely of prophylactic anticoagulation, without the use of complement inhibition. Both pregnancies ended successfully without thromboembolic complications.

We conclude that in pregnant patients with PNH and low-level hemolysis (i.e. LDH < 1.5 x ULN), management with close monitoring and prophylactic anticoagulation only, without use of complement inhibition, might be a reasonable strategy. More data to guide optimal management of pregnant women with PNH are needed.

## Introduction

Paroxysmal nocturnal hemoglobinuria (PNH) is a rare, clonal hematopoietic stem cell disorder characterized by hemolytic anemia and a high risk of arterial and venous thromboembolism. PNH is frequently accompanied by acquired bone marrow failure syndromes like aplastic anemia (AA) or myelodysplastic syndromes (MDS). Mechanistically, acquired somatic mutations in a clonal multipotent stem cell cause a defect in the glycosylphosphatidylinositol (GPI) anchor, which is a linker protein for multiple antigens such as CD55 and CD59, two complement regulator proteins, on the surface of various blood cells. Lack of these surface antigens makes erythrocytes vulnerable to complement-mediated attack, which results in intravascular, complement-mediated hemolysis [1]. 

The leading causes of morbidity and death in untreated PNH patients are thromboembolic complications, especially at atypical sites such as the mesenteric or the hepatic veins. The thromboembolic risk further increases during pregnancy. Historical data report high maternal mortality (up to 20%), mainly caused by venous thrombosis, and an increased fetal mortality [2]. Anticoagulant therapy was shown to reduce risk of thrombosis in PNH [3] and prophylactic anticoagulation is therefore recommended for pregnant patients with PNH [2, 4]. 

Complement inhibition has evolved as mainstay of PNH treatment, which changed the course of the disease from a life-threatening condition to a chronic illness [4]. Eculizumab, a monoclonal antibody against the complement protein C5, was the first complement inhibitor licensed for PNH [4]. One series of 75 pregnancies reported eculizumab as a safe and effective therapy option for PNH in pregnancy [5]. In this case series, no maternal deaths occurred and only 2 women developed thrombosis in the post-partum period [5]. However, the data also showed some degree of transport of the substance through the placenta [5]. 

In non-pregnant patients with PNH, complement inhibition is initiated when clinically relevant hemolysis occurs, defined by lactate dehydrogenase (LDH) ≥ 1.5 x ULN together with at least one PNH symptom such as anemia, dyspnea, dysphagia, abdominal pain, erectile dysfunction, hemoglobinuria or major adverse vascular events [6]. Indeed, eculizumab is only reimbursed at our institution when these criteria are met.

Whether this LDH cut-off should be also applied for pregnant patients with PNH is unclear. Here, we describe two pregnancies in two women with PNH and low-level hemolysis in whom we did not initiate complement inhibition but used thromboprophylaxis with low-molecular heparin (LMWH) only.

### Case 1

### Initial presentation

: In a 35-year-old woman, anemia and thrombocytopenia were incidentally detected in a routine blood draw during the first trimester of her first pregnancy (initial hemoglobin 10.2 g/dl, platelet count 64 G/l, leukocytes 3.9 G/l). Diagnostic workup including bone marrow cytology and immunophenotyping showed dysplasia suggestive but not decisive for MDS. Bone marrow cellularity was slightly hypercellular with signs of dysplasia of erythropoiesis and megakaryopoiesis. Cytogenetics and next generation sequencing of bone marrow revealed no evidence of pathologic abnormalities. Conservative management with close controls during pregnancy was chosen. In gestational week 28, peripheral blood parameters indicated low-level hemolysis, with hemoglobin 8.3 g/dl, reduced haptoglobin and slightly elevated LDH (256 U/l, ULN: 250 U/l). Platelet counts further declined to 44G/l. Coombs test was negative. A PNH clone with a granulocyte clone size of 34% was detected. The patient was asymptomatic, had no signs of bleeding or thrombosis and obstetric examination was unremarkable. There was no history of thrombosis or other relevant diseases.

### Management

**Fig. 1:**
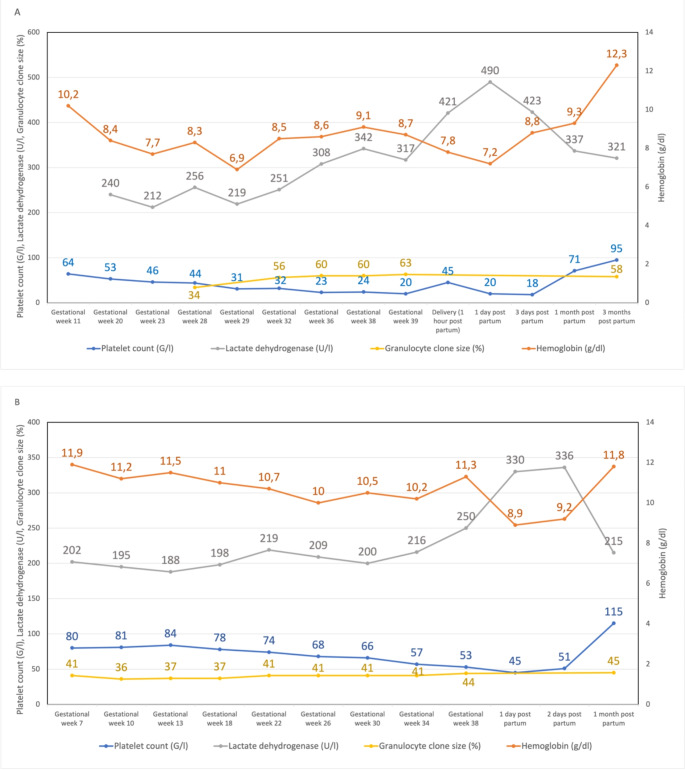
Hemoglobin concentration, platelet count, LDH concentration and granulocyte clone size (%) in 2 patients (case 1[**A**] and case 2[**B**]) with PNH in the setting of acquired bone marrow failure syndromes during pregnancy and the post-partum period. Both patients had regular visits at the outpatient hematology department and received prophylactic anticoagulation with low-molecular weight heparin (dose adjusted according to platelet counts). Immunosuppressive or complement inhibitory therapy were not required in our patients, as the course of their disease remained stable during the observation period

: Weekly checkups to monitor blood counts and hemolysis parameters were performed during pregnancy. Figure 1A shows laboratory parameters during pregnancy and the post-partum period. LDH constantly slightly increased during the course of pregnancy to a maximum of 342U/l in gestational week 38; however, as LDH levels remained < 1.5 x ULN complement inhibition was not started. Granulocyte clone size increased during the course pregnancy to 63% in gestational week 39. Platelet counts steadily decreased to a minimum of 20G/l in gestational week 39. Prophylactic anticoagulation with LMWH was prescribed and adjusted to platelet counts: We administered 40 mg of enoxaparin once daily when platelet counts were above 40 G/l, reduced the dose to 20 mg daily for platelet counts between 30 and 40 G/l, and withheld anticoagulation when platelet counts fell below 30 G/l.

Consequently, anticoagulation was stopped from gestational week 36 (platelet count: 23G/l) until delivery (platelet count in gestational week 39: 20G/l).

### Outcome

: A healthy boy was born via spontaneous vaginal delivery at gestational week 40 + 0. One unit of platelet concentrates and a total of 4 units of packed red blood cells were used to treat post-partum hemorrhage. Postpartum, we noted an increase in LDH levels, peaking at 490 U/l, which was attributed mainly to the delivery process [7, 8]. Consequently, eculizumab was not initiated, and indeed LDH decreased shortly after delivery (to 423U/l three days post-partum, 337U/l one month post-partum). Concomitantly, blood counts improved spontaneously steadily after delivery: One-month post-partum hemoglobin levels increased to 9.3 g/dl and platelet counts to 71G/l. Three months post-partum blood counts further improved to almost normal levels with hemoglobin 12.3 g/dl and platelet counts of 95 G/l (Fig. 1A). Prophylactic anticoagulation was resumed 10 days post-partum and continued beyond 6 weeks post-partum. Since PNH-granulocyte-clone size was > 50% (Fig. 1A) ongoing anticoagulation and a switch to oral anticoagulation was recommended to the patient. However, the patient refused such treatment and only consented to salicylic acid 50 mg once daily, starting 3 months postpartum. No thrombotic complications occurred during pregnancy and the postpartum period.

### Case 2

### Initial presentation

: A 31-year-old woman with a history of non-severe AA and PNH presented at our hematology outpatient department for counselling because of planned in-vitro fertilization (IVF). PNH associated with non-severe-AA was first diagnosed at age 27 following a routine blood count analysis. Bone marrow biopsy was hypocellular, with cellularity below 25% of normal. So far, no treatment for AA or PNH was required. The patient had stable blood counts (hemoglobin 14 g/dl, platelet count 83 G/l, leukocyte count 3.8 G/l), a granulocyte clone size of 43%, only minimal signs of hemolysis (reduced haptoglobin; LDH 264 U/l [ULN: 250 U/l]) and had no PNH associated clinical symptoms. Medication consisted of levothyroxine for hypothyroidism.

### Management

: The patient was informed about the increased maternal and fetal risk due to AA and PNH during pregnancy, including an increased risk of thrombosis, requirement of thromboprophylaxis and the possible need to start treatment for AA and/or PNH during pregnancy. Vaccinations against meningococcal infections were administered prior to IVF, in case the start of complement inhibition would have been necessary during pregnancy. Additionally, prophylactic dose LMWH (enoxaparin 40 mg once daily) was prescribed during hormonal stimulation for IVF and continued after successful embryo implantation throughout the pregnancy. We noted stable hemoglobin levels during the course of pregnancy (11.9 g/dl in gestational week 7, 11.3 g/dl in gestational week 38) and slowly declining platelet counts from 80G/l in gestational week 7 to a minimum of 53G/l in gestational week 38. Granulocyte clone size was 41% in gestational week 7 and stayed on a similar level throughout pregnancy (44% in gestational week 38). LDH levels stayed in the normal range throughout pregnancy (202U/l in gestational week 7, 216U/l in gestational week 34), and showed a slight increase up to 250U/l in gestational week 38. Consequently, as LDH stayed < 1.5 x ULN during the entire pregnancy, complement inhibitor therapy was not initiated and the patient received prophylactic anticoagulation only. Following mild vaginal bleeding in gestational week 10, the initial dose of 40 mg enoxaparin was reduced to 20 mg once daily. Subsequently, no further bleeding complications were reported. Course of laboratory parameters during pregnancy and post-partum is shown in Fig. 1B.

### Outcome

: A planned caesarean section was conducted at gestational week 39 + 0 without maternal or fetal complications, resulting in the birth of a healthy girl. One day post-partum, hemoglobin levels of 8.9 g/dl and platelet counts of 45G/l were recorded. LDH levels increased to a maximum of 336U/l two days post-partum. Thromboprophylaxis was reestablished after surgery and continued for 6 weeks post-partum. No thrombotic or bleeding complications occurred. Also, in this patient, after delivery blood counts improved spontaneously, resulting in hemoglobin levels of 11.8 g/dl, platelet counts of 115 G/L and a normalized LDH (215U/l) one-month post-partum. Granulocyte clone size one-month post-partum was stable (45%).

Both patients provided informed consent for the publication of these case reports.

## Discussion

Morbidity and mortality from thromboembolic events and other complications improved dramatically since the introduction of complement inhibitors for the treatment of PNH [9]. Complement inhibitor therapy is recommended for PNH patients with hemolysis and clinical symptoms indicative of high disease activity. A LDH cut-off of ≥ 1.5 x ULN is used for definition of elevated hemolysis in the non-pregnant setting. Whether the same LDH cut-off should be applied for pregnant patients with PNH is unclear. Identification of patients who can be securely managed without complement inhibition during pregnancy would be desirable to avoid the risk of meningococcal infections associated with such therapy.

Eculizumab, the first complement inhibitor licensed for PNH, has improved outcomes of pregnant PNH patients without reported safety concerns, despite its presence in 7 out of 22 cord-blood samples in one study [5]. Some guidelines suggest eculizumab for all pregnant PNH patients, if the granulocyte clone size is > 20%, even though data to support this cut-off are limited [10]. 

While complement inhibition is crucial for some pregnant PNH patients, it may not be necessary for those who do not clearly benefit. Side effects of complement inhibitor therapy include the risk for severe infections with gram-negative bacteria, although these complications are rare, particularly when adequate vaccinations are administered. In one of our patients (Case 2), PNH was diagnosed before pregnancy, enabling vaccination prior to conception. In contrast, in the first patient, PNH was diagnosed during pregnancy. Thus, if complement inhibitor therapy would have been started, vaccination (and/or antibiotic prophylaxis) during pregnancy would have been necessary.

Risk factors for thromboembolic events in the general PNH population include a history of thromboembolic events, a PNH granulocyte clone size of at least 30%, and LDH ≥ 1.5 x ULN with symptoms of high disease activity [11]. In pregnancy, disease activity often worsens after the second trimester due to increased terminal complement formation [5, 12]. This might enhance the risk of thromboembolic complications, adding to the already increased baseline of venous thromboembolism associated with pregnancy and puerperium.

In our two cases, LDH levels did not exceed 1.5 x ULN during pregnancy despite significant PNH clone sizes (Fig. 1A and B), and both patients had low platelet counts, possibly lowering thrombosis risk. Of note, both patients had PNH in the setting of bone marrow failure, and never experienced PNH related symptoms, suggesting that bone marrow failure syndrome was the prominent pathogenetic feature in our patients. Thus, a benefit from complement inhibitor therapy was considered less likely in our patients [13]. 

In non-pregnant PNH-patients, anticoagulation has been shown to reduce thrombotic risks in particular if the clone size was > 50% [3]. Moreover, prophylactic anticoagulation for pregnant women with PNH is recommended even in the setting of complement inhibition [2, 4]. Thus, we adopted an antithrombotic strategy using LMWH.

In conclusion, our cases suggest that pregnant PNH-patients with low-level hemolysis can be managed successfully without complement inhibitors using only prophylactic anticoagulation. However, close monitoring and initiation of complement inhibition in case of aggravating hemolysis is imperative. Yet more data are needed to define a threshold for initiating complement inhibition in pregnant PNH patients.

## Data Availability

No datasets were generated or analysed during the current study.

## References

[1] Hill A, DeZern AE, Kinoshita T, Brodsky RA (2017) Paroxysmal nocturnal haemoglobinuria. Nat Rev Dis Primers 3:17028. 10.1038/nrdp.2017.28 28516949 10.1038/nrdp.2017.28 PMC7879566

[2] Ray JG, Burows RF, Ginsberg JS, Burrows EA (2000) Paroxysmal nocturnal hemoglobinuria and the risk of venous thrombosis: review and recommendations for management of the pregnant and nonpregnant patient. Haemostasis 30:103–117. 10.1159/000022532 11014960 10.1159/000022532

[3] Hall C, Richards S, Hillmen P (2003) Primary prophylaxis with warfarin prevents thrombosis in paroxysmal nocturnal hemoglobinuria (PNH), blood 102. 3587–3591. 10.1182/blood-2003-01-0009 10.1182/blood-2003-01-0009 12893760

[4] Brodsky RA (2021) How I treat paroxysmal nocturnal hemoglobinuria. Blood 137:1304–1309. 10.1182/blood.2019003812 33512400 10.1182/blood.2019003812 PMC7955407

[5] Kelly RJ, Höchsmann B, Szer J, Kulasekararaj A, de Guibert S, Röth A, Weitz IC, Armstrong E, Risitano AM, Patriquin CJ, Terriou L, Muus P, Hill A, Turner MP, Schrezenmeier H (2015) Peffault De Latour, Eculizumab in pregnant patients with Paroxysmal Nocturnal Hemoglobinuria. N Engl J Med 373:1032–1039. 10.1056/NEJMoa1502950 26352814 10.1056/NEJMoa1502950

[6] Almeida AM, Bedrosian C, Cole A, Muus P, Schrezenmeier H, Szer J, Rosse WF (2017) Clinical benefit of eculizumab in patients with no transfusion history in the International Paroxysmal Nocturnal Haemoglobinuria Registry. Intern Med J 47:1026–1034. 10.1111/imj.13523 28608499 10.1111/imj.13523

[7] Neal JL, Lowe NK, Corwin EJ (2013) Serum lactate dehydrogenase profile as a retrospective indicator of uterine preparedness for labor: a prospective, observational study. BMC Pregnancy Childbirth 13:128. 10.1186/1471-2393-13-128 23759027 10.1186/1471-2393-13-128 PMC3687574

[8] Heimback DP, Prezyna AP (1960) Lactic dehydrogenase in pregnancy and the puerperium. Am J Obstet Gynecol 79:108–112. 10.1016/0002-9378(60)90369-0 14400671 10.1016/0002-9378(60)90369-0

[9] Hillmen P, Muus P, Röth A, Elebute MO, Risitano AM, Schrezenmeier H, Szer J, Browne P, Maciejewski JP, Schubert J, Urbano-Ispizua A, de Castro C, Socié G, Brodsky RA (2013) Long-term safety and efficacy of sustained eculizumab treatment in patients with paroxysmal nocturnal haemoglobinuria. Br J Haematol 162:62–73. 10.1111/bjh.12347 23617322 10.1111/bjh.12347 PMC3744747

[10] Kulasekararaj A, Cavenagh J, Dokal I, Foukaneli T, Gandhi S, Garg M, Griffin M, Hillmen P, Ireland R, Killick S, Mansour S, Mufti G, Potter V, Snowden J, Stanworth S, Zuha R, Marsh J, Committee BSH (2024) Guidelines for the diagnosis and management of adult aplastic anaemia: a British Society for Haematology Guideline. Br J Haematol 204:784–804. 10.1111/bjh.19236 38247114 10.1111/bjh.19236

[11] Höchsmann B, Peffault de Latour R, Hill A, Röth A, Devos T, Patriquin CJ, Chou W-C, Jain D, Zu K, Wu C, Lee JW (2023) Risk factors for thromboembolic events in patients with paroxysmal nocturnal hemoglobinuria (PNH): a nested case-control study in the International PNH Registry. Ann Hematol 102:2979–2988. 10.1007/s00277-023-05402-3 37668788 10.1007/s00277-023-05402-3 PMC10567964

[12] Derzsy Z, Prohászka Z, Rigó J, Füst G, Molvarec A (2010) Activation of the complement system in normal pregnancy and preeclampsia. Mol Immunol 47:1500–1506. 10.1016/j.molimm.2010.01.021 20181396 10.1016/j.molimm.2010.01.021

[13] Babushok D.V. (2021) When does a PNH clone have clinical significance? Hematol Am Soc Hematol Educ Program 2021:143–152. 10.1182/hematology.2021000245 10.1182/hematology.2021000245 PMC8791108 34889408

